# VITCOMIC2: visualization tool for the phylogenetic composition of microbial communities based on 16S rRNA gene amplicons and metagenomic shotgun sequencing

**DOI:** 10.1186/s12918-018-0545-2

**Published:** 2018-03-19

**Authors:** Hiroshi Mori, Takayuki Maruyama, Masahiro Yano, Takuji Yamada, Ken Kurokawa

**Affiliations:** 1 Genome Evolution Laboratory, Center for Information Biology, National Institute of Genetics, Mishima, 411-8540 Japan; 2 Department of Biological Information, Graduate School of Bioscience and Biotechnology, Tokyo Institute of Technology, 2-12-1 M6-3, Ookayama, Meguro-ku, Tokyo, 152-8550 Japan

**Keywords:** 16S rRNA gene, Microbial community, Metagenomics, Taxonomic composition

## Abstract

**Background:**

The 16S rRNA gene-based amplicon sequencing analysis is widely used to determine the taxonomic composition of microbial communities. Once the taxonomic composition of each community is obtained, evolutionary relationships among taxa are inferred by a phylogenetic tree. Thus, the combined representation of taxonomic composition and phylogenetic relationships among taxa is a powerful method for understanding microbial community structure; however, applying phylogenetic tree-based representation with information on the abundance of thousands or more taxa in each community is a difficult task. For this purpose, we previously developed the tool VITCOMIC (VIsualization tool for Taxonomic COmpositions of MIcrobial Community), which is based on the genome-sequenced microbes’ phylogenetic information. Here, we introduce VITCOMIC2, which incorporates substantive improvements over VITCOMIC that were necessary to address several issues associated with 16S rRNA gene-based analysis of microbial communities.

**Results:**

We developed VITCOMIC2 to provide (i) sequence identity searches against broad reference taxa including uncultured taxa; (ii) normalization of 16S rRNA gene copy number differences among taxa; (iii) rapid sequence identity searches by applying the graphics processing unit-based sequence identity search tool CLAST; (iv) accurate taxonomic composition inference and nearly full-length 16S rRNA gene sequence reconstructions for metagenomic shotgun sequencing; and (v) an interactive user interface for simultaneous representation of the taxonomic composition of microbial communities and phylogenetic relationships among taxa. We validated the accuracy of processes (ii) and (iv) by using metagenomic shotgun sequencing data from a mock microbial community.

**Conclusions:**

The improvements incorporated into VITCOMIC2 enable users to acquire an intuitive understanding of microbial community composition based on the 16S rRNA gene sequence data obtained from both metagenomic shotgun and amplicon sequencing.

**Electronic supplementary material:**

The online version of this article (10.1186/s12918-018-0545-2) contains supplementary material, which is available to authorized users.

## Background

Sequence analysis of the 16S rRNA gene-based amplicon is widely used to determine structures of microbial communities [1, 2]. To understand such structures from a phylogenetic viewpoint, it is helpful to have a combined representation of the taxonomic composition of microbial communities and the phylogenetic relationships among taxa [3, 4]. These relationships can be represented by a phylogenetic tree, yet abundance information on thousands or more taxa in the community is difficult to represent simultaneously in a tree [5, 6]. To address this issue, we previously developed the tool VITCOMIC (VIsualization tool for Taxonomic COmpositions of MIcrobial Community) [7, 8]. VITCOMIC compares user-provided 16S rRNA gene sequences with reference 16S rRNA gene sequences obtained from genome-sequenced microbes and identifies the nearest relative of each submitted sequence. VITCOMIC then renders a visualization of the overall taxonomic composition of the sample with indications of sequence identity between the reference and sample sequences. Because VITCOMIC yields a combined representation of the taxonomic composition of a sample and the phylogenetic relationships among taxa, this tool has been adopted by many researchers [9–11].

Substantive improvements in VITCOMIC became necessary to address several issues related to the analysis of 16S rRNA gene-based microbial communities, noted as follows. (i) Public databases contain many 16S rRNA gene sequences from uncultured taxa [12], but VITCOMIC uses 16S rRNA gene sequences exclusively from genome-sequenced prokaryotes as references. (ii) The genomes of most microbes contain two or more copies of 16S rRNA genes [13, 14], but VITCOMIC does not normalize the bias of this taxonomic abundance that results from differences in 16S rRNA gene copy number among taxa. (iii) The number of sequences for each sample has increased significantly because of the rapid technological developments related to massively parallel DNA sequencing [15], but the calculation speed of VITCOMIC has not kept pace with data growth because VITCOMIC uses BLAST [16] for sequence identity searches. (iv) The use of 16S rRNA gene sequences obtained by shotgun metagenomic sequencing eliminates any bias that might result from the initial PCR amplification of 16S rRNA genes [17, 18], but the extraction of 16S rRNA gene sequences from such sequencing data based on sequence similarity searches against reference databases is problematic because some 16S rRNA gene sequences in public databases are contaminated by sequences from other genes (e.g., tRNA and 23S rRNA genes) [18]. To address these issues, we developed VITCOMIC2, which enables users to acquire an intuitive understanding of microbial community composition based on sequence data for the 16S rRNA gene obtained from both metagenomic shotgun and amplicon sequencing.

## Implementation

### Construction of a high-quality 16S rRNA gene sequence reference database including uncultured taxa

A high-quality 16S rRNA gene sequence reference database that includes uncultured taxa was constructed using 16S rRNA gene sequences obtained from the Ribosomal Database Project (RDP) [12]. The 1,345,732 16S rRNA gene sequences of Bacteria and Archaea in RDP (release 11, update 2) were retrieved using the following parameters: Strain = Both, Source = Both, Size ≥1200, and Quality = Good. We obtained these sequences with three different file formats: multiply aligned fasta files, unaligned fasta files, and GenBank files. To eliminate contaminating tRNA gene sequences, internal transcribed spacer sequences (ITSs), and 23S rRNA gene sequences from this reference database, we performed a search of the 1,345,732 sequences with BLAST+ [19] (version 2.2.27; −max_target_seqs 100 and -parc_identity 97) against the previous VITCOMIC 16S rRNA gene sequence reference database [20]. This previous database contains only 16S rRNA gene sequences for which the 5′-end was adjusted to six bases before the 8F primer motif sequence (TTGATCCT) [21] and the 3′-end, adjusted to the end of the anti-Shine–Dalgarno sequence (CACCTCCTTN) [22]; therefore, the 5′- and 3′-end trimming of each sequence for optimization of VITCOMIC2 was easily performed using the sequence identity search results. After trimming the 1,345,732 16S rRNA gene sequences, we eliminated (i) 476 sequences for which the length was abnormally long (> 1660 bp) or short (< 1120 bp) according to the distribution of sequence length (Additional file 1), and (ii) 1159 sequences that contained ≥10 bases of homopolymer.

Because the RDP database contains many nearly identical sequences, we performed genus-level sequence clustering using USEARCH [23] version 6.0.307 (−-cluster_smallmem --id 0.94, −-query_cov 0.9, and --target_cov 0.9). Since USEARCH/UCLUST version 6 uses a greedy algorithm, the order of input sequences is very important [23]. Therefore, before conducting sequence clustering, we have sorted sequences whose origins at the genus-level taxonomy were not designated as “unclassified” by their sequence lengths. Then, “unclassified” sequences were sorted by their sequence lengths, and added on the end of the sequence file. By sorting sequences as described above, in most cases, we can avoid the “unclassified” sequences in the RDP database become a representative sequence of a cluster. The resulting 63,956 representative sequences of clusters were examined to determine whether any were PCR chimeric sequences using UCHIME [24] (version 6.0.307 de novo mode and reference mode). In the UCHIME reference mode, gold.fa sequence data [25] were used as the reference. We discarded the 706 representative sequences that were flagged by both the de novo and reference modes of UCHIME as chimeric sequences. In addition, we discarded the 34,273 representative sequences whose origins at the genus-level taxonomy were designated as “unclassified”. Consequently, we obtained a database that consisted of 28,977 high-quality 16S rRNA gene sequences (Additional file 2 and Additional file 3).

### Allocation of references in VITCOMIC2

In the previous version of VITCOMIC, VITCOMIC visualized overall phylogenetic composition of a sample in one circular diagram using 16S rRNA gene sequences of genome sequenced prokaryote species as reference [7]. However, the number of genome sequenced prokaryote species are rapidly grown. In addition, the uncultured taxa whose genomes are not sequenced yet are also important to describe microbial communities. To include many reference sequences in VITCOMIC2, we need to separate a VITCOMIC circular diagram to two types of circular diagrams representing overall phylogenetic composition of a microbial community based on phylum composition and detailed phylogenetic composition in each phylum of the community based on genus composition. Since the separation of phylum and genus composition in different circular diagrams often cause difficulty to understand overall phylogenetic composition of a microbial community, we completely replaced a Perl and PostScript based static circular diagram in VITCOMIC to JavaScript Raphael library based interactive circular diagrams in VITCOMIC2.

**Fig. 1:**
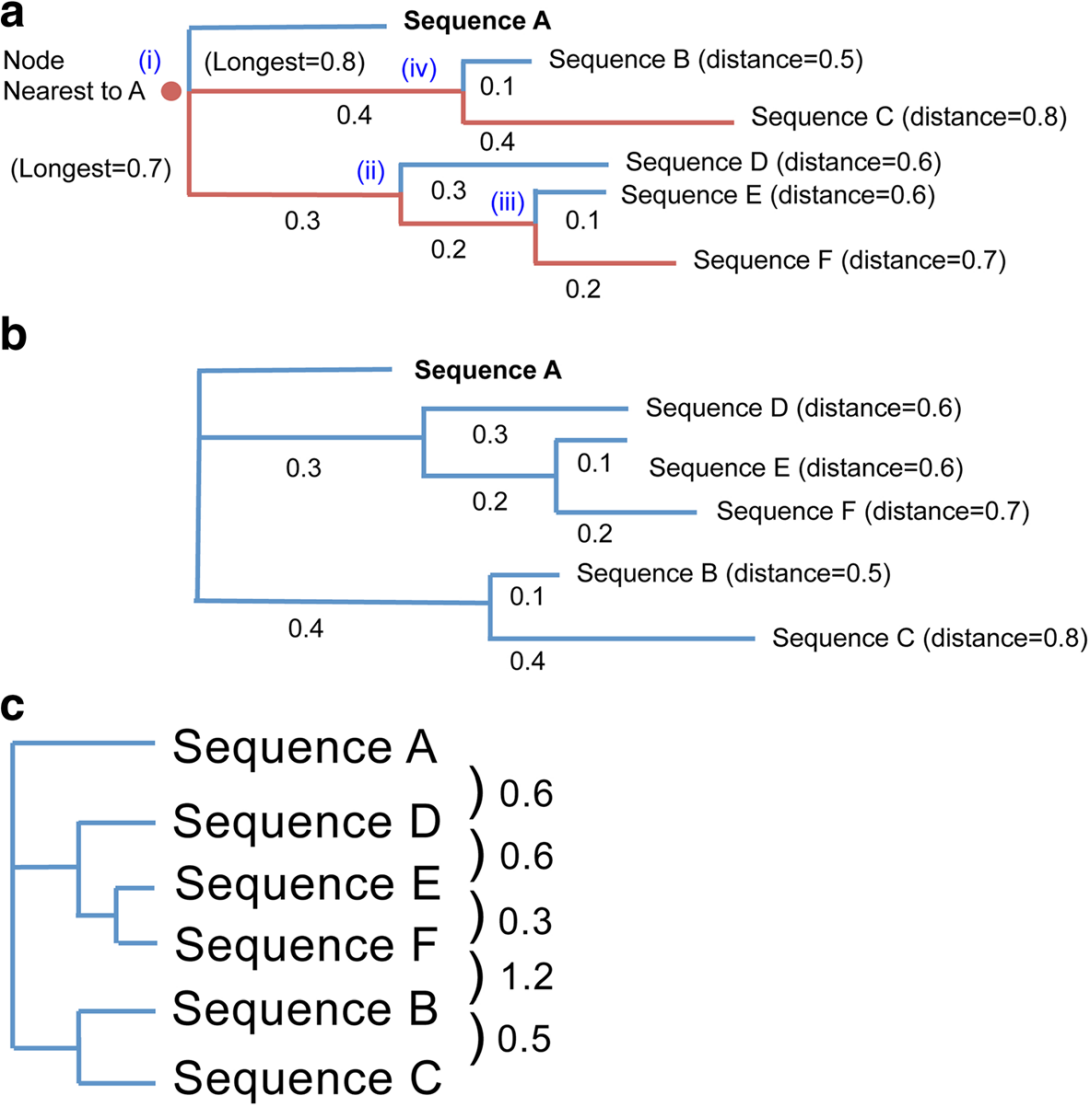
Schematic diagram of the method used to determine the order of sequences in a circular diagram. (**a**) The original phylogenetic tree that contains six sequences. Sequence A represents the initial sequence chosen for subsequent tree topology scanning. The order of the six sequences in the phylogenetic tree was determined as follows. Relative to sequence A, sequence C is the farthest, sequence B is the second farthest, sequence F is the third farthest, sequence E is the fourth farthest, and sequence D is nearest. (**b**) A rotated phylogenetic tree based on the order of sequences determined as in (**a**). (**c**) The order of, and phylogenetic distances between, the sequences that were determined as in (**a**)

VITCOMIC2 constructs a circular diagram representing overall phylogenetic composition of a microbial community based on phylum composition with the following procedure. (i) In each phylum, 30 sequences were randomly chosen from the high-quality 16S rRNA gene sequence database. As the phylum Proteobacteria is extraordinarily diverse [26], we randomly chose 30 sequences from every class of Proteobacteria. For any phylum having < 30 sequences, all available sequences were chosen. (ii) We thus obtained 1184 sequences from 44 phyla/classes. To calculate phylogenetic distances among them, the 1184 sequences were aligned using MAFFT [27] version 6.864b with default parameters. After constructing a multiple sequence alignment, genetic distances between sequences were calculated using the dnadist program in PHYLIP [28] version 3.69 with Kimura’s two-parameter model of base substitution [29]. The phylogenetic tree was constructed using the neighbor-joining method in the neighbor program of PHYLIP version 3.69. (iii) To construct the circular diagram from the phylogenetic tree, we randomly chose one sequence from Gammaproteobacteria to use as the initial sequence for subsequent tree topology scanning. The tree topology scanning is our concise method to represent tree topology (distance and cladogram) information in one line as follows. The sequence that was most distantly related to the initial sequence was identified by comparing the phylogenetic distances between the initial sequence and every other sequence (Fig. 1). The second most distantly related sequence was identified in the peripheral clade that contained the most unrelated sequence, and this process was reiterated to generate an ordered list of related sequences. (iv) The position on the circular diagram of each sequence in the list was determined based on its phylogenetic distance to its nearest relative. (v) Each phylum/class position in the circular diagram was determined by calculating an average position among sequences that belong to that phylum/class.

To understand the detailed taxonomic composition in each phylum, VITCOMIC2 also constructs a circular diagram that represents the genus composition of the sample as described by the following example. (i) The phylogenetic tree within each of the 44 phyla/classes was constructed by using all sequences belonging to each phylum/class in the high-quality 16S rRNA gene sequence database, i.e., the same method that was used for the phylum example. (ii) The same method was again used to construct the circular diagram within each of the 44 phyla/classes with a random choice of one sequence to serve as the initial sequence. If all the sequences derived from the same genus belonged to a single cluster, then the genus position in the circular diagram was the cluster position. Conversely, when the sequences derived from the same genus belonged to more than two clusters, the genus position in the circular diagram was determined by calculating an average position among the clusters.

### A rapid sequence identity search using graphics processing units (GPUs)

**Fig. 2:**
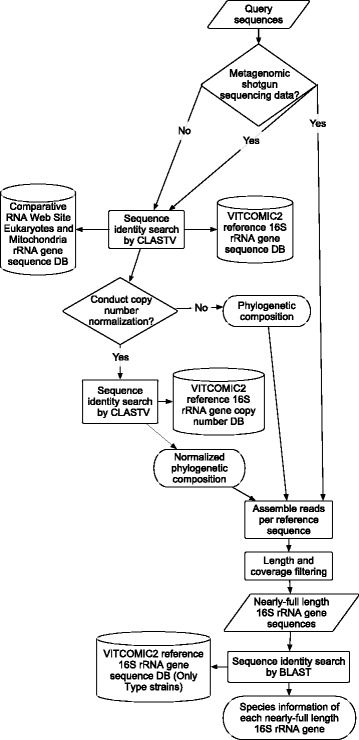
VITCOMIC2 overall workflow. The workflow is shown with the following symbols: parallelogram, sequence data; cylinder, sequence database; square, analysis; diamond, conditional branch; ellipse, output file

The overall workflow of VITCOMIC2 for processing data from user-uploaded 16S rRNA gene amplicon sequencing and metagenomic shotgun sequencing is described in Fig. 2. The inference of the nearest relative for each query sequence in VITCOMIC2 was performed with a modified version of CLAST [30], a GPU-based high-speed nucleotide sequence identity search tool that we developed. We made the following modifications to CLAST to enable us to specifically search 16S rRNA gene sequences. (i) We completely separated the processes of database indexing and alignment calculation. Because VITCOMIC2 does not update the reference sequence database frequently, pre-indexing the reference database is more efficient for daily calculations. (ii) We changed the method used to estimate the amount of GPU memory consumed. In the original CLAST program, total nucleotide lengths of sequences were used to estimate the amount of the GPU memory consumed. In the modified version of CLAST for VITCOMIC2, referred to as CLASTV, the total number of sequences was used because there was little variation in the lengths of the query or database sequences. We compared the calculation speed between VITCOMIC2 and VITCOMIC by using 1,119,519 16S rRNA gene sequences sequenced by the 454 GS FLX sequencing platform obtained from Turnbaugh et al. [31] as query sequences. For VITCOMIC we used one process of BLAST+ with the parameters (−evalue 1e-08 and -num_alignments 100) and for VITCOMIC2 we used one process of CLASTV with the thresholds (identity ≥80% and alignment length ≥ 50 bp). The speed test was carried out on a machine with a Xeon X5670 2.93 GHz CPU and a Tesla C2050 GPU.

### Normalizing differences in 16S rRNA gene copy number among taxa

Although some microbial genomes contain only one copy of the 16S rRNA gene, most microbes contain two or more copies [13]. Therefore, the taxonomic abundance of a microbial community directly inferred from 16S rRNA gene sequence abundance is generally biased because of differences in 16S rRNA gene copy number among taxa [32]. To avoid this bias in VITCOMIC2, we implemented the following method to normalize 16S rRNA gene copy number differences among taxa. Previous work suggested that phylogenetically cross-related taxa tend to have a similar number of 16S rRNA gene copies in the genome [33]. To obtain 16S rRNA gene copy number information for the phylogenetically related taxa of the user sequence, VITCOMIC2 conducts a sequence identity search using CLASTV against a 16S rRNA gene copy number reference database (16S rRNA-CN DB) of the genome-sequenced strains, which was constructed as follows. (i) The 16S rRNA gene sequences for each genome-sequenced strain were identified using RNAmmer [34] against the genomic sequence obtained from the NCBI Genome Database [35] in April 2014. (ii) One 16S rRNA gene sequence was randomly sampled per species because there are only small sequence differences among 16S rRNA genes within a single genome and across genomes within a single species [13]. We obtained 1505 16S rRNA gene sequences, which thus comprised the 16S rRNA-CN DB (Additional file 4). The sequence most related to the user sequence was then identified based on the results of a sequence identity search against the 16S rRNA-CN DB. VITCOMIC2 assumes that the 16S rRNA gene copy number is identical between the genomes of the user sequence and the nearest sequence in the 16S rRNA-CN DB.

We used a leave-one-out approach to validate the accuracy of the VITCOMIC2 16S rRNA gene copy number inference method. To identify the most related sequence, we randomly chose one sequence from the 16S rRNA-CN DB and conducted a sequence identity search with BLAST+ (−max_target_seqs 100) against this database (lacking the query sequence). Comparing the 16S rRNA gene copy number between the genomes of the query sequence and the nearest sequence, we calculated the error ratio for inferring the 16S rRNA gene copy number for all 1505 sequences from the 16S rRNA-CN DB.

### Taxonomic composition analysis using the 16S rRNA gene sequences extracted from metagenomic shotgun sequencing data

To evaluate the specificity of the VITCOMIC2 extraction method for 16S rRNA gene sequences from metagenomic shotgun sequencing data, we compared the VITCOMIC2 method with a common extraction method using BLAST+ against the original RDP sequence database. Because we needed to eliminate the effect of different sensitivities between the two databases (i.e., the effect of different taxonomic coverage between the two databases on this comparison), we designated the 1,345,256 sequences in the VITCOMIC2 16S rRNA gene sequence reference database before conducting homopolymer filtering and 94% identity clustering as the reference sequence database. For comparison, we used soil metagenomic shotgun sequencing data (DRR001464) [18], as sequenced by the Illumina Genome Analyzer IIx sequencer. For the alternative sequence extraction method, the 47,195,934 sequences from DRR001464 were searched against the original RDP sequence database using BLAST+ with E-value <1e-8. The sequences extracted from the VITCOMIC2-based method and the original RDP sequence database-based method were searched against the NCBI-nt database [36] obtained as of July 2014 using BLAST+ with E-value <1e-4; each hit was manually verified with regard to gene function to evaluate whether the specific extracted sequence was indeed a 16S rRNA gene.

### Validation of accuracy of the genus assignments and the 16S rRNA gene copy number normalization by using mock community metagenomic data

We used the Illumina HiSeq metagenomic sequencing data of Singer et al. [37] (read length = 150 bp) of a mock community of 26 genome-sequenced strains (23 genera) for the validation of accuracy of the genus assignments and the 16S rRNA gene copy number normalization.

The molarity of the DNA of each species in the mock community was previously measured [37]. In addition, the 16S rRNA gene copy number in the genome of each species is described in the RefSeq genome sequence database [36]. Therefore, the theoretical abundance of 16S rRNA gene sequences from each species in the mock community metagenomic sequencing data was calculated by multiplying the molarity of the DNA of each species and the 16S rRNA gene copy number in the genome. We compared the genus compositions of communities resulting from (i) the theoretical composition based on the molarity of the DNA of each species, (ii) the theoretical composition based on multiplying the molarity of the DNA of each species and the 16S rRNA gene copy number of the genome, (iii) 16S rRNA gene sequence-based composition calculated by VITCOMIC2, and (iv) 16S rRNA gene sequence-based composition calculated by VITCOMIC2 with 16S rRNA gene copy number normalization.

In addition, we compared genus composition inference accuracies of the Singer et al. mock community sequencing data among VITCOMIC2 and three other software (MAPSeq version 1.2 [38], SortMeRNA version 2.1b [39], and RiboTagger version 0.8.1 [40]). To separate the reference sequence databases differences and the genus composition inference accuracies of tools, we used the VITCOMIC2 high-quality 16S rRNA gene reference sequence database as a reference sequence database for MAPSeq and SortMeRNA. In the VITCOMIC2, MAPSeq, and SortMeRNA results, we only used hits with the threshold (identity ≥94%, alignment length ≥ 100 bp, and top hit) for genus assignments. In the RiboTagger analysis, we specified a parameter “-r v4”.

### Species inference based on the nearly full-length 16S rRNA gene sequence reconstructed from metagenomic shotgun sequencing data

The ecological niches of taxa are sometimes different among species in the same genera [41]. However, the partial 16S rRNA gene sequences obtained from the metagenomic or amplicon sequencing analyses by the short read sequencers usually do not contain enough information for species identification [42]. Therefore, we developed a function to reconstruct nearly full-length 16S rRNA gene sequences from metagenomic shotgun sequencing data in VITCOMIC2. The reconstruction workflow is as follows. (i) Based on CLASTV results in VITCOMIC2, 16S rRNA gene reads from metagenomic shotgun sequencing data that are assigned to the same reference sequence are extracted and assembled by MEGAHIT version 1.1.1 with default parameters. (ii) After contig length (≥800 bp) and read coverage (≥10) filtering, nearly full-length 16S rRNA gene sequences are obtained. (iii) BLAST-based sequence identity searches are conducted using the nearly full-length 16S rRNA gene sequences against the type strain 16S rRNA gene sequences in the VITCOMIC2 16S rRNA gene sequence reference database. (iv) The names of species are assigned to the nearly full-length 16S rRNA gene sequences when ≥97% sequence identities are observed in the BLAST-based sequence identity searches from (iii). The accuracy of the above species inference method was evaluated by using Illumina HiSeq metagenomic data of the mock community [37].

### VITCOMIC2 web system

The VITCOMIC2 web server [43] can accept either a fasta or a fastq file of one 16S amplicon or metagenomic shotgun sequencing sample with read length ≥ 100 bp as the query. Users can choose whether to include 16S rRNA gene copy number normalization and nearly full-length 16S rRNA gene sequence reconstruction. After uploading an input file, VITCOMIC2 conducts analyses as described in Fig. 2. (i) VITCOMIC2 conducts a CLASTV-based sequence identity search against the VITCOMIC2 high-quality 16S rRNA gene reference sequence database with the threshold (identity ≥80% and alignment length ≥ 100 bp, and top hit). (ii) To exclude rRNA gene sequences of eukaryotes and mitochondria, VITCOMIC2 also conducts CLASTV-based sequence identity searches against the 18S rRNA gene and rRNA gene sequences of eukaryotes and mitochondria obtained from Comparative RNA Web Site version 2 [44] with the same threshold. (iii) If users didn’t choose to conduct 16S rRNA gene copy number normalizations and the nearly full-length 16S rRNA gene sequence reconstructions, a genus composition tab-separated text file by setting identity threshold ≥94% and VITCOMIC2 phylum and genus composition figures are created by using information of CLASTV hits which were in (i)'s analysis but were not in (ii)'s analysis. (iv) In case of conducting 16S rRNA gene copy number normalizations, VITCOMIC2 conducts a CLASTV-based sequence identity search against the VITCOMIC2 16S rRNA-CN DB after (i) and (ii) analyses. Using the VITCOMIC2 16S rRNA-CN DB information, each hit in (i)'s analysis is normalized as described above section. After that, a normalized genus composition tab-separated text file and VITCOMIC2 phylum and genus composition figures are created. (v) In case of conducting nearly full-length 16S rRNA gene sequence reconstructions from metagenomic shotgun sequencing data, MEGAHIT assembling and BLAST-based sequence identity search against the type strain 16S rRNA gene sequences in VITCOMIC2 reference sequence database are performed as described above section. When the VITCOMIC2 analysis is finished, VITCOMIC2 sends an Email to the user with a download link to the results which include a VITCOMIC2 visualization result, a genus composition text file, reconstructed nearly full-length 16S rRNA gene sequences fasta files and species assignment result text files, and a text file for the VITCOMIC2 Comparison. Users can conduct multiple-sample comparisons by uploading VITCOMIC2 analysis result files for selected samples to the VITCOMIC2 Comparison web server [45].

## Results and discussion

### Description of the VITCOMIC2 circular diagram

**Fig. 3:**
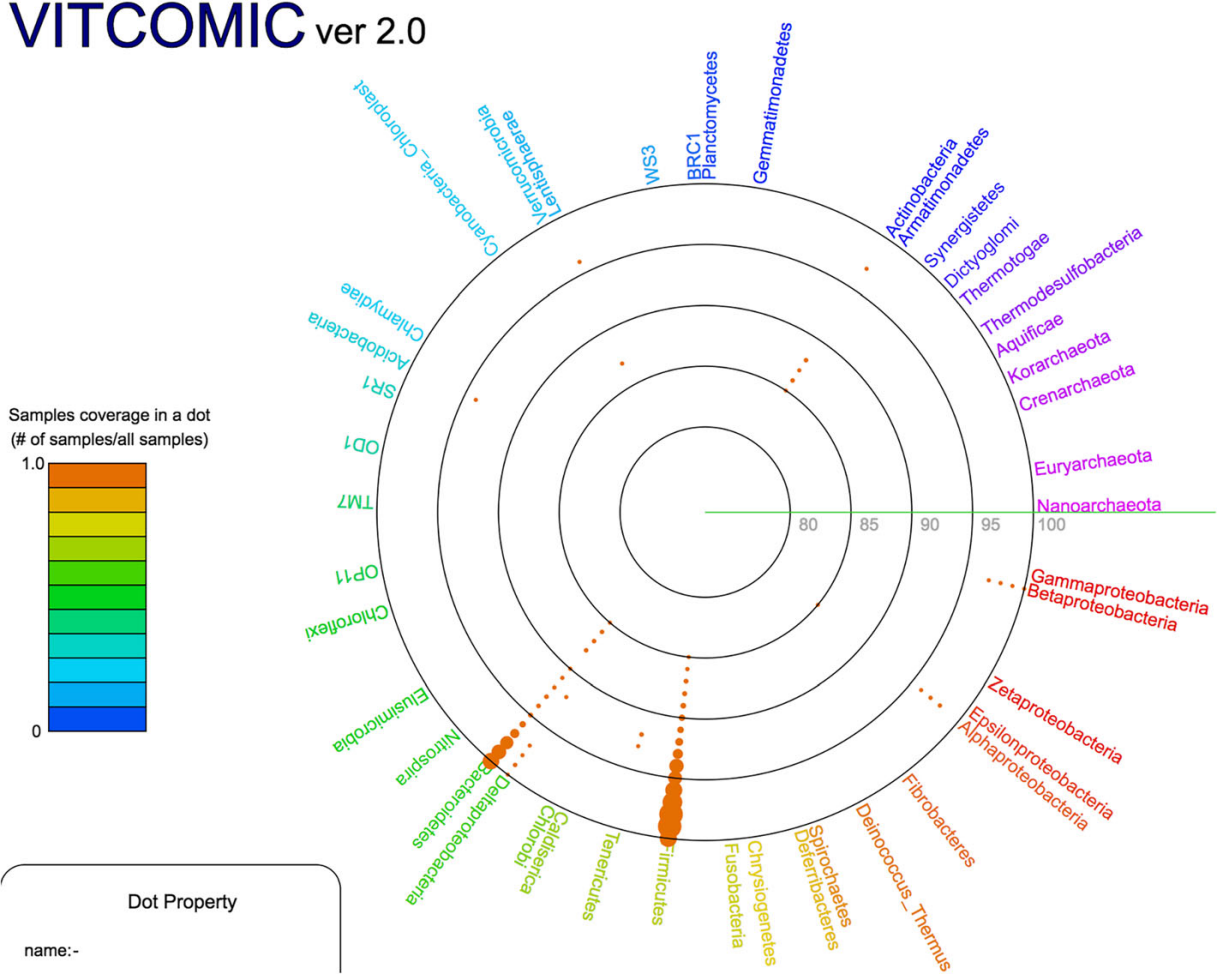
Example of VITCOMIC2 representation of the phylum composition and associated phylogenetic relationships. Phylum composition of a sample from the Turnbaugh et al. study [31]. For VITCOMIC2, the basic representation strategy does not differ from that of the original VITCOMIC, although the following additional information is represented in VITCOMIC2. (i) Sizes of dots represent the relative abundance of reads in the sequence cluster. (ii) When users select a dot, they obtain information on the number of sequences that the cluster contains. (iii) Genus composition of the phylum can be viewed by selecting the phylum name

**Fig. 4:**
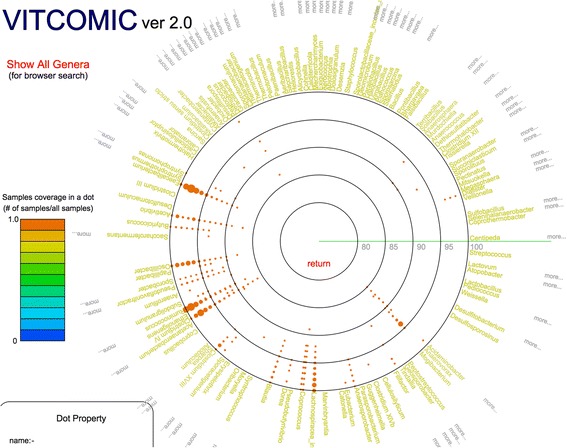
Example of VITCOMIC2 representation of the genus composition and associated phylogenetic relationships. Genus composition of a samples from the Turnbaugh et al. study [31]. When two or more genera are located at the same location, these genera names can be seen by selecting the “more” button for specific genera, or by selecting the “Show All Genera” button to view all genera at once

**Fig. 5:**
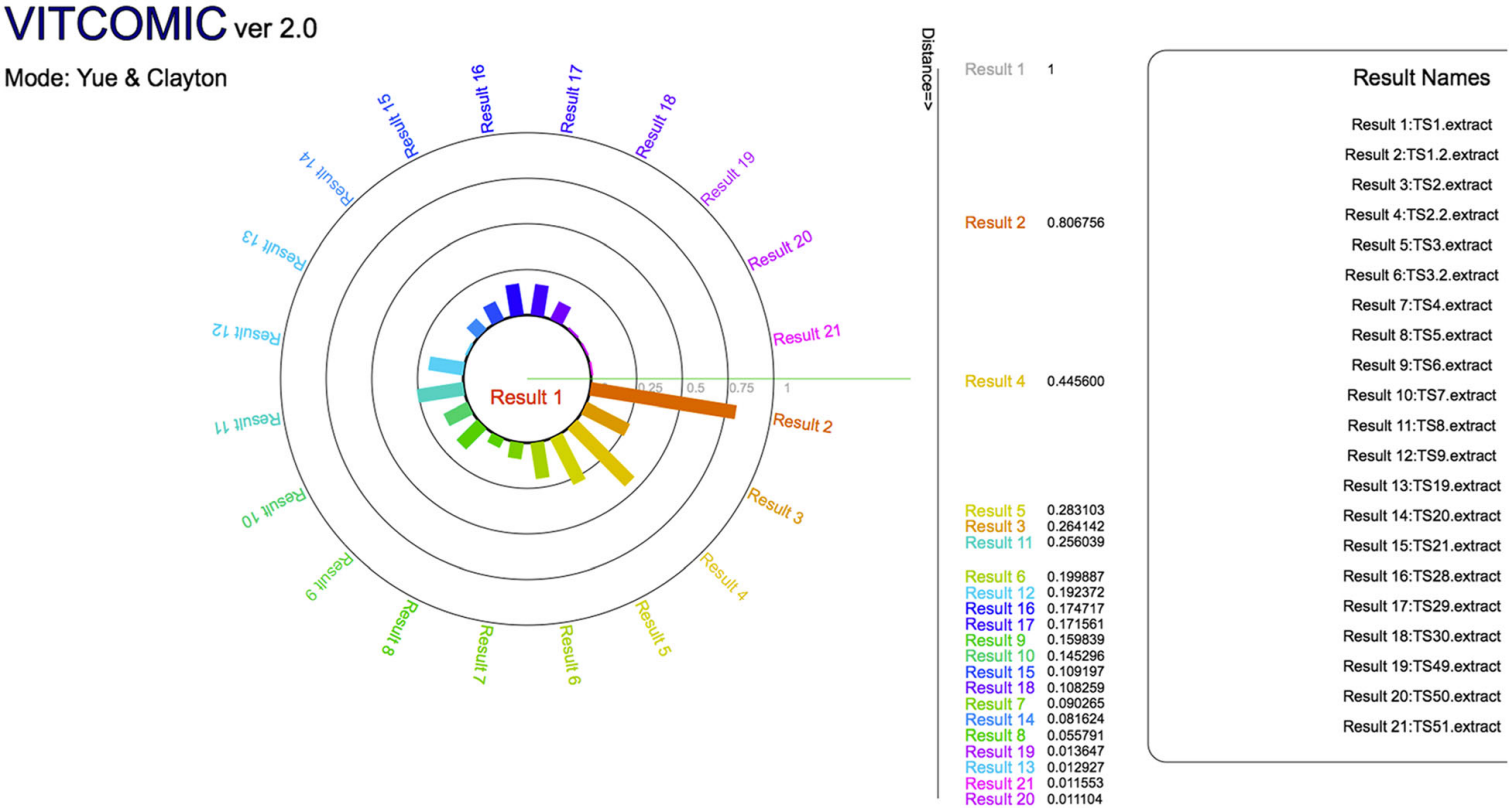
Example of a VITCOMIC2 statistical sample comparison. The sequence cluster composition among samples is used to calculate Jaccard similarity, Pearson correlation, and the Yue and Clayton theta similarity coefficients. The Yue and Clayton theta similarity coefficient between a particular sample and the other 21 samples from the Turnbaugh et al. study [31] is shown. Users can change the sample for comparison by selecting the sample name

VITCOMIC2 represents the phylum and genus composition of samples and their phylogenetic relationships using a circular diagram (Fig. 3 and Fig. 4). VITCOMIC2 Comparison calculates several statistical coefficients for pairwise community comparison (Jaccard similarity, Pearson correlation, and Yue and Clayton theta similarity coefficients [7]) (Fig. 5). In VITCOMIC2 Comparison, to conduct an accurate statistical comparison between samples, only those query sequences that match a reference database sequence with ≥94% identity are used. These features of the VITCOMIC2 circular diagram help the user understand microbial community composition more intuitively.

In the previous version of VITCOMIC, to fit the order of reference taxa on the VITCOMIC circular diagram with the known phylogenetic relationships among taxa, we had to manually rotate the reference phylogenetic tree. For a phylogenetic tree with 28,977 branches, this rotation was time consuming. In addition, we have little knowledge of the phylogenetic relationships for the uncultured taxa. Therefore, we developed a reference taxa allocation method to construct the VITCOMIC2 circular diagram automatically. Application of this method to the previous VITCOMIC reference data confirmed that the order of the species remained the same as that determined with the manual rotation-based circular diagram (Spearman rank correlation coefficient = 0.98), thereby validating the VITCOMIC2 reference taxa allocation method.

### Sensitivity and speed of VITCOMIC2 as compared with VITCOMIC

The time needed to analyze the 1,119,519 16S rRNA gene amplicon sequences [31] using VITCOMIC2 was 20 min, much faster than the 5 h 31 min needed for VITCOMIC. This vast improvement in speed was achieved despite the fact that VITCOMIC only contains only 601 16S rRNA gene reference sequences and no normalization calculation function for determining the 16S rRNA gene copy number. The number of reads whose identity with the reference sequences was ≥80% was 1,106,215 for VITCOMIC and 1,112,958 for VITCOMIC2. The average top hit identity of these reads was 92.3% for VITCOMIC and 98.0% for VITCOMIC2. For the taxonomic composition analysis based on 16S rRNA gene sequences, unassigned sequences are usually neglected for the analysis, and this is especially the case for metagenomic shotgun sequencing, for which it is difficult to confirm that the unassigned sequences are definitely not 16S rRNA genes. Therefore, it is crucial to maximize the sensitivity of the taxonomy assignment and reduce the fraction of unassigned sequences so that the taxonomic composition of microbial communities can be appropriately inferred. To maintain sensitivity but reduce calculation time, we removed phylogenetically redundant sequences in the reference database by conducting sequence clustering. Using CLASTV and the phylogenetically non-redundant high-quality 16S rRNA gene sequence database including uncultured taxa, VITCOMIC2 greatly improved the sensitivity and reduced the calculation time as compared with VITCOMIC.

### Validation of the VITCOMIC2 16S rRNA gene copy number normalization method

Additional file 5 presents the results of the leave-one-out accuracy validation for the VITCOMIC2 normalization method for 16S rRNA gene copy number. More than 90% of the examined sequences were accurately inferred with regard to the 16S rRNA gene copy number within a twofold error. Strains for which the inferred 16S rRNA gene copy number differed from the real copy number by more than threefold were phylogenetically less-studied taxa or taxa that have very different copy numbers compared to closely related strains (Additional file 6).

**Fig. 6:**
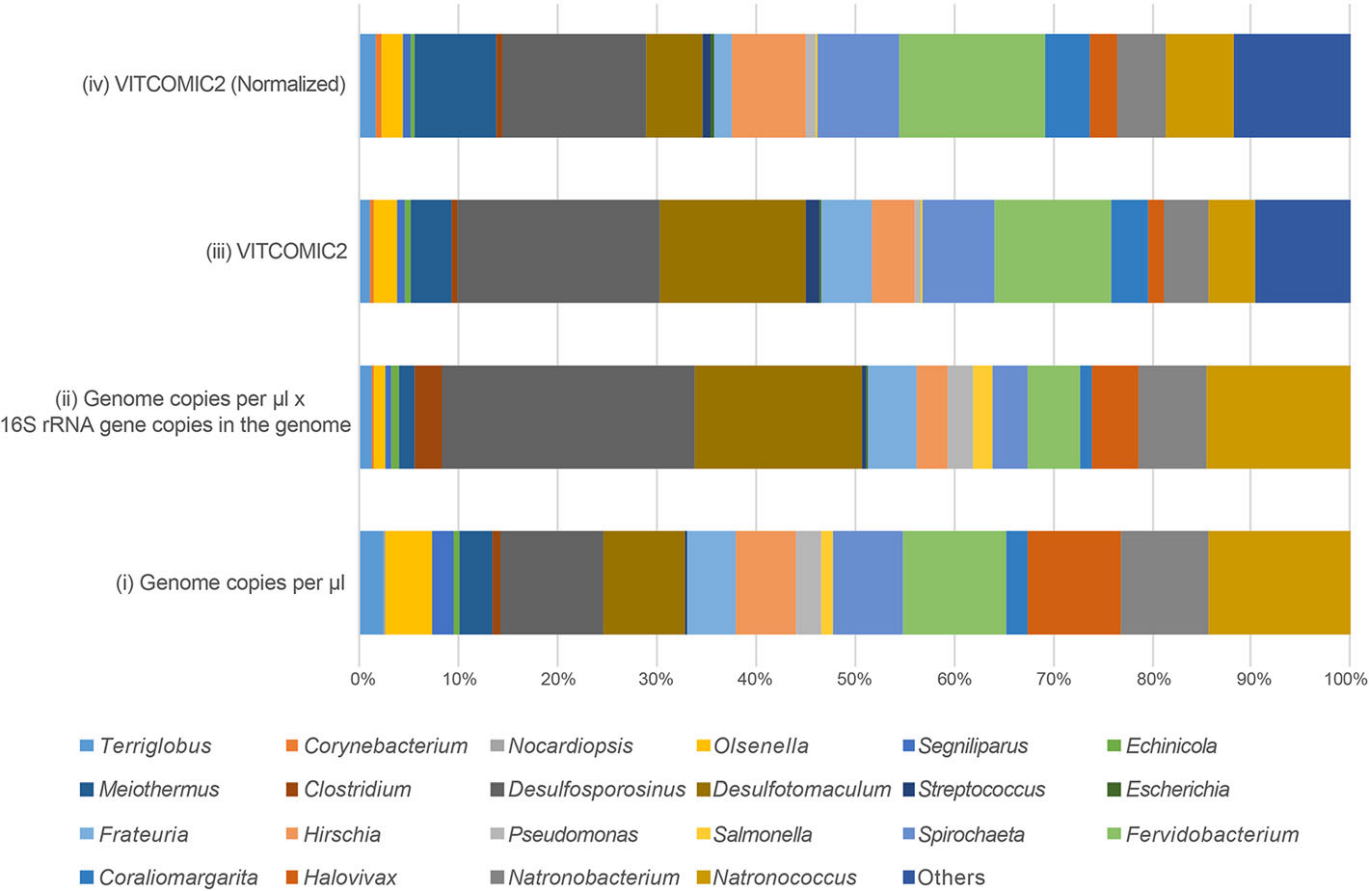
Comparison of genus composition of a mock community among four samples. VITCOMIC2 (Normalized) indicates the 16S rRNA gene sequence-based genus composition as calculated by VITCOMIC2 after conducted the 16S rRNA gene copy number normalization. (i), (ii), (iii), and (iv) correspond to sample descriptions in the main text: Implementation Section: “Validation of accuracy of the genus assignments and the 16S rRNA gene copy number normalization by using mock community metagenomic data”

Figure 6 presents the two theoretical genus compositions (i and ii) and two VITCOMIC2-based genus compositions (iii and iv) of mock metagenomic sequencing data from Singer et al. [37]. We removed the *Thermobacillus* abundances data from all compositions, as the authors reported that there was contamination by *Thermobacillus*. VITCOMIC2-based genus compositions contain “Others” (other genera), which correspond to genera that did not exist in the original mock community. Because almost all of the genera in “Others” are closely related to the 23 genera in the mock community, “Others” may be artifacts that were produced from the combination of sequencing errors in metagenomic shotgun reads and misassignments of genera by VITCOMIC2. The Pearson correlation coefficients was 0.79 between (ii) the theoretical genus composition (based on multiplying the molarity of the DNA of each species and the 16S rRNA gene copy number of the genome) and (iii) the 16S rRNA gene sequence-based genus composition (calculated by VITCOMIC2); in comparison, the Pearson correlation coefficient was 0.59 between (i) the theoretical genus composition (based on the molarity of the DNA of each species) and (iv) the 16S rRNA gene sequence-based genus composition (calculated by VITCOMIC2 with the 16S rRNA gene copy number normalization). These Pearson correlation coefficients suggest the validity of the VITCOMIC2 method of genus assignment and 16S rRNA gene copy number normalization.

Additional file 7 presents a comparison result of genus composition inference accuracies of the Singer et al. mock community sequencing data among four tools. Spearman correlation coefficients of genus compositions between the theoretical composition and each tool’s inferred composition are described. Except for RiboTagger, because three tools used same reference sequence database in this analysis, the Spearman correlation coefficient differences indicates the genus inference accuracy differences caused by algorithmic differences among three tools. Among four tools, VITCOMIC2 exhibits highest genus composition inference accuracy. This comparison result also suggests that the validity of the VITCOMIC2 method of genus assignment.

### Validation of specificity of VITCOMIC2 with metagenomic shotgun sequencing data

Additional file 8 presents the results of the specificity analysis of the VITCOMIC2 16S rRNA gene sequence extraction method as compared with an alternative sequence extraction method using BLAST+ and the original RDP sequence database. Although the latter method extracted more sequences than did the VITCOMIC2 method, most of the specifically extracted sequences in the two methods were not 16S rRNA gene sequences (e.g., they included tRNA genes, ITS regions, and 23S rRNA genes). Because the evolutionary histories differ between 16S rRNA genes and other genes/regions, such non-16S rRNA gene sequences may confound the analysis of microbial community composition and diversity. Although some of the 16S rRNA gene sequences were specifically obtained with the method using the original RDP database, these sequences represented only a small percentage (0.4%) compared with that of false positive sequences (5.6%) that were specifically identified with the original RDP database. In addition, the calculation time needed for the sequence extraction method using the BLAST+ and original RDP sequence database was 10 h 14 min using a computer with four CPU cores. By contrast, the corresponding calculation time for VITCOMIC2 was 82 min. This result indicates that VITCOMIC2 specifically and rapidly extracts 16S rRNA gene sequences from metagenomic shotgun sequencing data.

### Species inference based on the nearly full-length 16S rRNA gene sequence reconstructed from metagenomic shotgun sequencing data

Additional file 9 presents the results of reconstruction and species assignment of the nearly full-length 16S rRNA gene sequence reconstructed from mock metagenomic sequencing data [37]. All five sequences showed 100% sequence identity with the original 16S rRNA gene sequence of the strain. Because this reconstruction method does not conduct the reference-based mapping assembly, some of the sequences contained ITS and intergenic regions located near the 5′ and 3′ ends of the 16S rRNA gene. Although it is difficult to know why only the 16S rRNA gene sequences from four minor species were able to be reconstructed, one possibility is that the Illumina sequencing platform-dependent sequence errors in some specific sequence patterns [46] may make assembly difficult in the 16S rRNA gene sequences of major (i.e., high read coverage) species.

## Conclusions

We developed VITCOMIC2 as a means to rapidly analyze the phylogenetic composition of microbial communities based on data from the 16S rRNA gene sequences obtained from both metagenomic shotgun and amplicon sequencing with normalization of 16S rRNA gene copy number differences among genomes. The results from each VITCOMIC2 analysis are graphically visualized with two levels of taxonomic rank to facilitate understanding of taxonomic features at the level of the whole community and of the specific taxonomic groups. In addition to phylogenetic composition analyses, the combination of a highly accurate reference 16S rRNA gene sequence database in VITCOMIC2 and a rapid sequence identity search by CLASTV made it possible to reconstruct nearly full-length 16S rRNA gene sequences and assign species from metagenomic shotgun sequencing data. This concise and rapid analysis method with the interactive visualization system of VITCOMIC2 will undoubtedly contribute to an intuitive understanding of the phylogenetic composition of microbial communities for diverse users.

## Additional files

Additional file 1: Length distribution of 16S rRNA gene sequences in the RDP database. The length distribution of 1,345,732 16S rRNA gene sequences in the RDP database is shown after trimming of the 5′ and 3′ ends. (PDF 815 kb)

Additional file 2: List of RDP IDs for the 28,977 high-quality 16S rRNA gene sequences in VITCOMIC2. (XLS 3610 kb)

Additional file 3: List of phylum names for the 28,977 high-quality 16S rRNA gene sequences in VITCOMIC2. (XLS 21 kb)

Additional file 4: List of strain names in the 16S rRNA gene copy number reference database. a. For each strain, a RefSeq ID is shown of the representative replicon that contains 16S rRNA gene copies. b. Class name of the strain in the phylum Proteobacteria or phylum name of the strain in other phyla is shown. (XLS 210 kb)

Additional file 5: Distribution of the leave-one-out accuracy validation for estimating 16S rRNA gene copy number with the VITCOMIC2 copy number normalization method. The value 0 on the horizontal axis indicates the number of species that can be estimated based on the correct 16S rRNA gene copy number by applying the VITCOMIC2 copy number normalization method. (PNG 83 kb)

Additional file 6: List of the strains for which the inferred 16S rRNA gene copy number differed by more than threefold as compared with the real copy number. (DOC 24 kb)

Additional file 7: Correlation coefficients of genus composition of the Singer et al. mock community between theoretical composition and four tools’ composition. (XLS 63 kb)

Additional file 8: Number of assigned sequences for several sequence categories using the two different reference sequence databases. a. “Others” indicates that sequences contain more than two categories of sequences (e.g., 16S rRNA genes and tRNA genes). (XLS 25 kb)

Additional file 9: Species assignment results from reconstructed nearly full-length 16S rRNA gene sequences from the metagenomic shotgun sequencing data of a mock community. (XLS 61 kb)

## Abbreviations

16S rRNA-CN DB: 16S rRNA gene copy number reference database
CLAST: CUDA implemented large-scale alignment search tool
CLASTV: CLAST for VITCOMIC2
GPU: Graphics Processing Unit
ITS: internal transcribed spacer.
RDP: Ribosomal Database Project
VITCOMIC: VIsualization tool for Taxonomic COmpositions of MIcrobial Community
