# Supplementary figures and images for: VITCOMIC2: visualization tool for the phylogenetic composition of microbial communities based on 16S rRNA gene amplicons and metagenomic shotgun sequencing
Source: BMC Syst Biol. 2018 Mar 19;12(Suppl 2):30. doi: 10.1186/s12918-018-0545-2 (PMC5861490; doi:10.1186/s12918-018-0545-2)

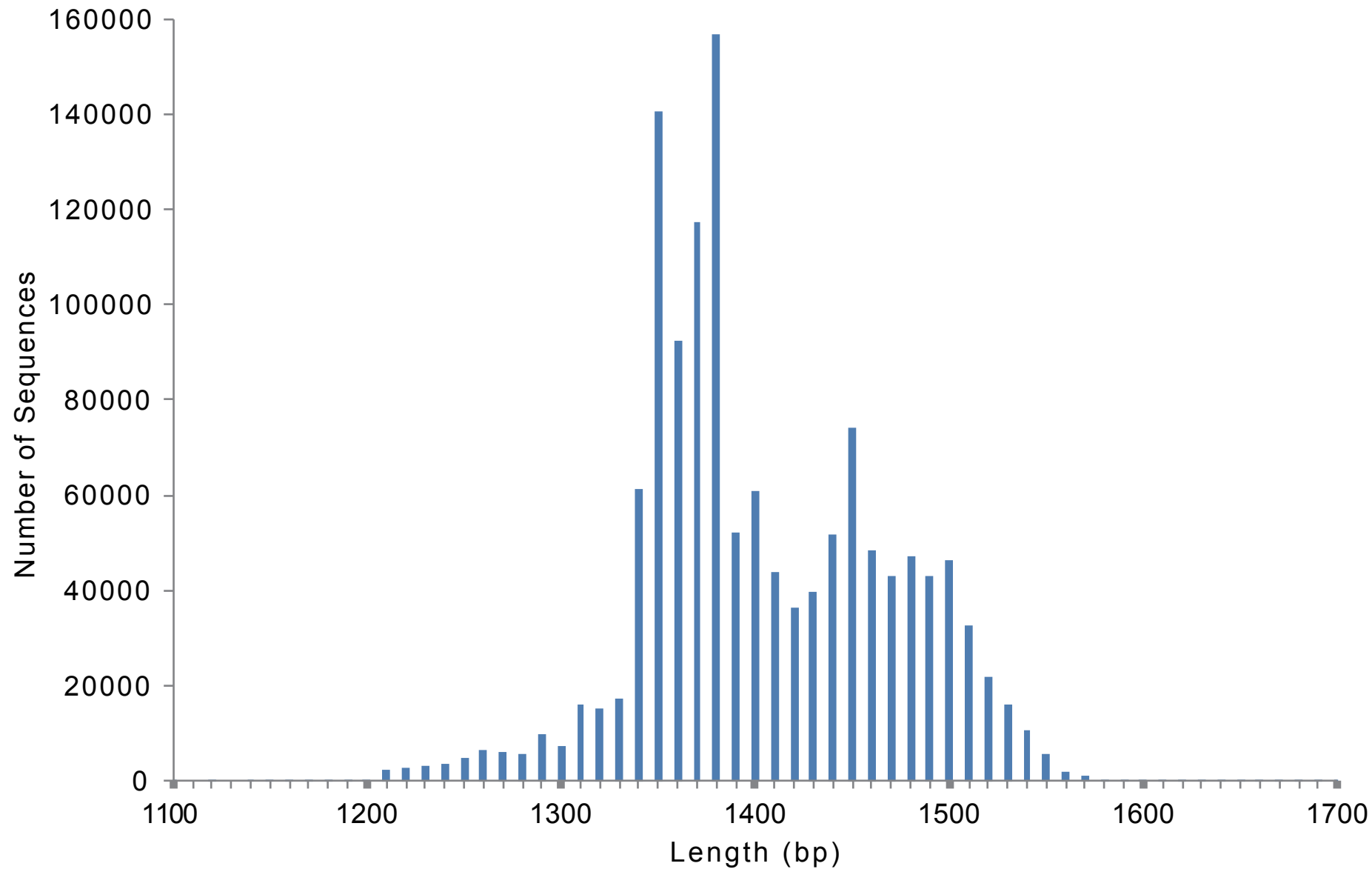

Supplement: Supplementary file 1 — Length distribution of 16S rRNA gene sequences in the RDP database. The length distribution of 1,345,732 16S rRNA gene sequences in the RDP database is shown after trimming of the 5′ and 3′ ends. (PDF 815 kb) [file 12918_2018_545_MOESM1_ESM.pdf]

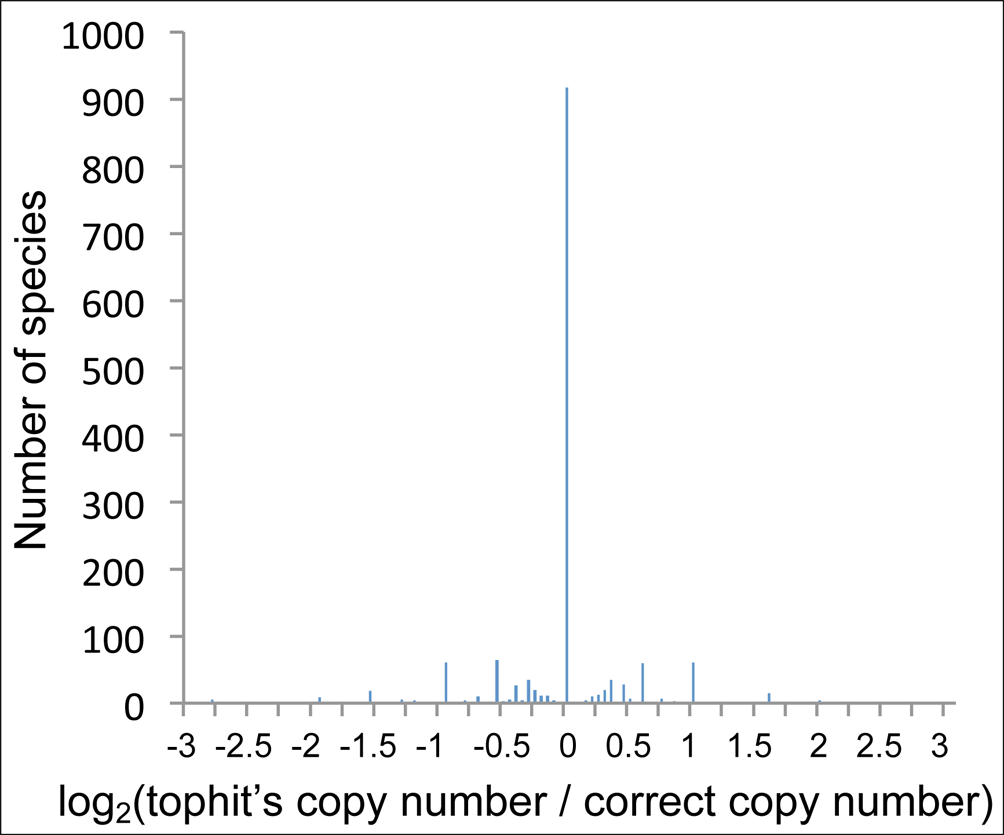

Supplement: Supplementary file 5 — Distribution of the leave-one-out accuracy validation for estimating 16S rRNA gene copy number with the VITCOMIC2 copy number normalization method. The value 0 on the horizontal axis indicates the number of species that can be estimated based on the correct 16S rRNA gene copy number by applying the VITCOMIC2 copy number normalization method. (PNG 83 kb) [file 12918_2018_545_MOESM5_ESM.png]
